# Between Ideal and Actual Care: Patients’ and Family Carers’ Experiences of Cancer Care Relationships

**DOI:** 10.3390/ejihpe16050058

**Published:** 2026-04-23

**Authors:** Claudia Venuleo, Serena Miccoli, Alessia Petrachi, Tiziana Marinaci

**Affiliations:** Department of Human and Social Sciences, University of Salento, 73100 Lecce, Italy; serenamiccoli65@gmail.com (S.M.); alessiapetrachi80@gmail.com (A.P.); tiziana.marinaci@unisalento.it (T.M.)

**Keywords:** cancer patients, family carers, ideal care relationship, perceived care experience, physician-patient relationship, semi-structured interviews, thematic analysis

## Abstract

Research on how patients and family carers experience their relationships with physicians and healthcare staff is limited, particularly regarding the gap between ideal expectations and actual care. This study explored patients’ and carers’ perceptions of the ideal care relationship, their lived experiences, and factors shaping discrepancies between expectations and reality. A total of 143 individual, face-to-face semi-structured interviews (mean age = 56.7 ± 13.2; 61.4% women) were conducted with 57 cancer patients and 86 family carers in outpatient oncology clinics in Southern Italy. Participants were recruited through purposive sampling and interviewed separately, with carers recruited as an independent group. Transcripts were analysed using Thematic Analysis of Elementary Contexts (TAEC), a mixed-methods approach combining qualitative and quantitative techniques. Methodological rigor and trustworthiness were ensured in line with COREQ reporting guidelines. Four thematic clusters emerged: “Variability in the experience,” “The ideal care relationship,” “Waiting times and delays in care,” and “The luck of being cared for by a good physician.” Oncology care experiences emerge as inherently ambivalent: supportive in interactions with clinicians, yet tension-laden due to systemic and organizational constraints. These findings suggest that strengthening patient- and family-centered care requires both relational improvements and organizational interventions aimed at reducing waiting times, enhancing care integration across fragmented pathways, and improving continuity of care.

## 1. Introduction

A serious illness such as cancer—still often culturally associated with a “death sentence” (Moser et al., 2021)—entails profound fear, anxiety, and uncertainty, and has substantial social consequences for patients and family carers (Guan et al., 2021; Mattock et al., 2025). Family members often take on practical, emotional, and social support roles when caring for a patient with cancer, which carry considerable physical, emotional, and social burden and can deeply affect everyday life (Ferrell & Wittenberg, 2017; Grycuk et al., 2022; Soh et al., 2025; Tan et al., 2018; Thompson et al., 2024).

Research increasingly conceptualizes cancer-related distress as a shared and interdependent experience, with patients and family carers showing parallel patterns of psychological adjustment over time (Badr et al., 2014; Pruchno et al., 2009). Recent qualitative research further highlights that caregivers actively shape patients’ experiences by negotiating communication and meaning within clinical encounters, thereby influencing both access to and quality of care (Sterponi et al., 2024). These relational practices underscore that cancer care is not solely an individual experience but a socially embedded process, in which patients and family carers jointly navigate complex healthcare systems. From this perspective, cancer—like other chronic illnesses—can be understood as a “family affair” (Dieperink et al., 2018), in which meanings, emotions, and coping strategies are co-constructed within relational contexts.

The quality of the healthcare relationship—encompassing dimensions such as communicative clarity, emotional support, empathy, and responsiveness to patients’ and family carers’ needs—plays a crucial role in shaping how cancer is experienced and made sense of by both patients and family carers. These relational aspects have been consistently associated with treatment adherence, engagement with care, health outcomes, and overall satisfaction across healthcare settings (Di Blasi et al., 2001; Ohana & Mash, 2015; de Waard et al., 2018; Venuleo et al., 2019). Recent evidence in oncology confirms and extends these findings, showing that unmet informational, emotional, and relational needs—which remain highly prevalent among both patients and caregivers (Lewandowska et al., 2021; Yang et al., 2021; T. Wang et al., 2018)—together with organizational and systemic barriers related to care coordination, access, and waiting times (OECD, 2025) are associated with increased caregiver and patient burden and poorer psychological adjustment, as well as lower propensity to engage with and attend follow-up care (Hill et al., 2023; Lambert & Girgis, 2017).

While previous research has extensively highlighted the importance of relational aspects of care and their impact on patient satisfaction with care and ability to cope with cancer (Luhar, 2025; Prip et al., 2018), fewer studies have examined how patients’ subjective experiences these relationships, and even less attention has been paid to the perspectives of family carers. In many healthcare contexts, clinical practices continue to prioritize the patient as the sole focus of care, often positioning family members as “outsiders” (Coyne, 2013) or relegating them “to the sidelines” of care processes (Coyne et al., 2017). As a result, family carers’ needs related to information, communication, emotional support, and involvement in care decisions frequently remain unmet (Chong et al., 2023; Liu et al., 2022).

Attending to users’ subjective experiences is widely regarded as a cornerstone of the humanization of care (Bombard et al., 2018; Cheraghi et al., 2017) particularly in contexts characterized by high emotional and existential burden such as oncology care where uncertainty, fear, waiting, and fragmented care trajectories are well-documented features of patients’ and family carers’ experiences (Lopes et al., 2025). Despite this, existing research has rarely examined these experiences by jointly considering patients and family carers, nor has it sufficiently addressed the discrepancy between ideal expectations of care and lived experiences within real-world healthcare systems shaped by organizational, temporal, and relational constraints.

Against this backdrop, the present study aims to address these gaps by exploring patients’ and family carers’ experiences of healthcare responsiveness, defined as the extent to which health systems meet users’ expectations regarding the non-clinical dimensions of care (World Health Organization, 2000), with a specific focus on relational and experiential aspects. Specifically, the study examines how patients and family carers represent what the care relationship should be like, how it is actually experienced, and how discrepancies between ideal and actual care are explained and made sense of, focusing on the interpretative frameworks and key thematic structures through which participants construct and interpret their experiences.

## 2. Materials and Methods

Traditionally, users’ views of healthcare have been assessed through patient satisfaction surveys, which require respondents to express their experiences within categories predefined by researchers (DiCicco-Bloom & Crabtree, 2006). Moreover, satisfaction studies often report high levels of satisfaction that do not necessarily indicate the absence of problems, but may instead reflect gratitude, social desirability bias, the enactment of a passive patient role, or beliefs about the legitimacy of one’s expectations and reluctance to voice dissatisfaction (Schneider & Palmer, 2002).

To overcome these limitations, this study adopted a mixed-methods design, integrating qualitative data collection with quantitative text analysis techniques. Specifically, qualitative semi-structured in-depth interviews were used to elicit patients’ and family carers’ narratives, allowing them to articulate what matters to them in their relationship with the healthcare system and to express perceived care needs in their own terms. Quantitative data-driven lexical analysis was then applied to identify patterns of co-occurrence and the underlying semantic and interpretative structures through which patients and family carers construct meanings around the care relationship.

From a constructionist perspective, narratives are relevant not because they offer a direct account of events, but as sites where meanings are produced and negotiated, revealing the interpretative frames through which experiences are understood and evaluated (Gergen, 1985). Accordingly, the analysis was aimed at capturing these meaning-making processes, rather than providing a purely descriptive account of care experiences.

Methodological rigor was ensured by adhering to established criteria for qualitative research trustworthiness, in line with COREQ reporting guidelines. This was operationalized through systematic analytical procedures, including independent coding by multiple researchers, comparison of interpretations, and consensus-based resolution of discrepancies during data analysis.

### 2.1. The Interview

The study was conducted in the outpatient clinics of the oncology department of a hospital in Southern Italy, within a collaboration agreement between the Local Health Agency overseeing the hospital and the university department of the research team. The interviews were conducted by nine trained interviewers, all psychology graduates involved in the research project. The interviewers received preliminary training to ensure consistency in interview administration, focusing on study objectives, key themes, and methodological procedures (Jacob & Furgerson, 2012). The training included theoretical and practical components, such as familiarization with the interview guide, role-playing exercises, and discussion of potential challenges in the field, including managing participants’ emotional distress, avoiding directive questioning that could influence responses, and following the natural flow and rhythm of participants’ narratives. In line with a constructionist paradigm, interviewers adopted an active listening approach (Adhabi & Anozie, 2017), adapting questions in real time to encourage participants to articulate their experiences and meaning-making processes. This flexibility allowed narratives to be jointly constructed within the interview setting rather than constrained by rigid questioning (Riessman, 2008). Particular attention was paid to creating a welcoming atmosphere and exploring responses in depth (Kallio et al., 2016). To ensure consistency across interviewers, regular debriefing meetings were held during the data collection phase, allowing the team to align procedures and address emerging issues. Each interview was guided by four core questions (Table 1): three focused on the relationship with the physician and one on the relationship with healthcare staff more broadly. Participants were first asked to describe the key characteristics of a good doctor–patient relationship and then to reflect on their most recent hospital visit. Focusing on the last visit aimed to reduce recall bias and avoid selectively reporting particularly positive or negative experiences. Participants were subsequently asked to reflect on the factors they believed accounted for any discrepancies between their ideal and actual experiences. The fourth question addressed their overall experience with the healthcare staff encountered in the hospital. Finally, participants were invited to add any further comments.

Participants’ experience of the interview itself was explored through a closing question (e.g., “How do you feel at the end of this interview?”). To contextualise accounts, information on sex, age, and reason for attending the hospital was collected.

All interviews were conducted individually. Patients and family carers were interviewed separately, and the latter were recruited as an independent group within the same clinical setting.

Interviews lasted 10–20 min with additional time devoted to rapport building and post-interview debriefing. All interviews were conducted in a private room provided by the hospital, ensuring a confidential and comfortable setting. Sessions were audio-recorded and transcribed verbatim.

### 2.2. Recruitment and Procedure

A purposive sampling approach was used to recruit participants. Eligibility criteria required both patients with cancer and family carers to be aged 18 years or older. Individuals with known cognitive impairment (e.g., a diagnosis of Alzheimer’s disease or dementia, or current use of anti-dementia medication) were excluded.

The study was conducted in the outpatient clinics of the oncology department of a hospital in Southern Italy, within a collaboration agreement between the Local Health Agency overseeing the hospital and the university department of the research team. Recruitment began in March 2024 and lasted three and a half months. The final sample size was considered adequate as data collection reached thematic sufficiency, with no new relevant patterns emerging in relation to the research aims (Braun & Clarke, 2019).

The nine interviewers rotated in the hospital, covering at least three mornings and three afternoons per week. Potential participants were approached in the oncology ward waiting room, where the study was also advertised via poster. During an initial briefing, study aims, voluntary participation, anonymity, and audio-recording procedures were explained. To reduce potential emotional challenges, interview topics were outlined in advance, and interviews were conducted in a private room (Whiting, 2008). Participants were reminded of their right to skip questions or pause the interview. Although the exact number of individuals who declined participation was not systematically recorded, refusal was mainly due to lack of time or physical discomfort. Written informed consent was obtained from all participants prior to the interview, after they had received detailed information about the study aims, procedures, and their rights, including the right to withdraw at any time without consequences. Interviews were scheduled and conducted in a way that did not interfere with hospital routines or clinical activities. Participants were approached while waiting for their appointment, and interviews were either conducted before or after the visit, depending on patient preference and time availability.

All interviews were conducted in Italian, as it was the native language of participants, allowing them to express their experiences more naturally and in greater depth.

### 2.3. Participants

A total of 143 interviews were conducted (Mean age: 56.73 ± 13.19; women: 69.2%), including 57 with cancer patients (Mean age: 62.27 ± 11.15; women: 61.4%) and 86 with family carers (Mean age: 52.92 ± 13.19; women: 74.4%). All patients had received a diagnosis. Table 2 presents participants’ characteristics.

### 2.4. Data Analysis

Data were managed in compliance with confidentiality and anonymity requirements. All interviews were audio-recorded, transcribed verbatim, and anonymized by removing any potentially identifying information. Transcripts were stored in password-protected digital files accessible only to the research team. The final corpus was organized as a single text dataset. A mixed-methods approach combining qualitative and quantitative techniques was used to analyse the transcripts, aiming to map the main themes underpinning the narratives. To this end, Automatic Co-occurrence Analysis for Semantic Mapping (ACASM; Gennaro & Salvatore, 2023; Salvatore et al., 2017) was applied to the entire corpus using T-LAB software, version 10 (Lancia, 2023). ACASM is a context-sensitive automated textual analysis method that detects word co-occurrences within a defined context unit (e.g., a paragraph). Its premise is that meanings are not inherent in isolated words but emerge from their co-occurrence within discourse dynamics (Salvatore et al., 2012; Venuleo, 2013). Unlike frequency-based approaches, ACASM examines co-occurrences at the level of Elementary Context Units (ECUs), which are small text segments representing meaning units. Lexical forms within ECUs are reduced to their corresponding lemmas (e.g., “go”, “goes”, “gone” → “to go”), and lemma co-occurrences are used to construct a semantic network. The resulting data matrix includes ECUs as rows and lemmas as columns, with binary values indicating lemma presence or absence, following T-LAB’s standard unsupervised clustering procedure. Table 3 summarizes the dataset characteristics.

The T-LAB Thematic Analysis of Elementary Contexts (TAEC) was then used to identify recurring themes in participants’ narratives. This unsupervised clustering method groups ECUs with similar lexical patterns, revealing emergent semantic structures (Lancia, 2023). After clusters were identified, a second data matrix (Clusters × Lemmas) was generated for interpretation, containing absolute lemma frequencies and their Chi^2^ values indicating the degree to which each lemma characterizes a cluster.

The most representative ECUs, ranked by lexical proximity to each cluster, together with the distribution of statistically significant lemmas, were used as the empirical basis for cluster interpretation. In operational terms, this process allows the interpretation of clusters as configurations of meaning emerging from recurrent patterns of co-occurrence. For instance, lexical items such as “communication,” “information,” and “answer” tend to co-occur with verbs such as “to ask,” “to explain,” and “to understand,” within ECUs including participants’ accounts of asking for clarification during medical visits and descriptions of difficulties in understanding clinical explanations. When these lexical patterns are considered in relation to their distribution across the corpus and their co-occurrence structures, they can be interpreted as expressing a broader thematic area related to information exchange and the need for clarity within the care relationship.

Clusters were then interpreted by identifying their shared thematic core, reflecting specific ways of representing experiences or expressing viewpoints (Gennaro et al., 2012; Marinaci et al., 2025; Venuleo & Guacci, 2014). Three authors familiar with ACASM and TAEC independently proposed descriptive labels using a double-blind procedure; discrepancies were resolved through consensus.

Finally, Correspondence Analysis (CA) was applied to examine associations between clusters and respondent role (patient vs. family carer), socio-demographic variables (sex, age), and contextual conditions (first visit, treatment, follow-up). Test-values exceeding ±1.96 were considered statistically significant (*p* < 0.05).

## 3. Results

### 3.1. Semantic Cores

Cluster analysis identified four main semantic cores (Table 4).

These clusters represent distinct semantic configurations associated with different dimensions of the care experience of patients and caregivers, including variability in care quality, expectations regarding the ideal therapeutic relationship, experiences of waiting and organizational delays, and positive encounters with healthcare professionals. They are described below with reference to the most representative Elementary Context Units (ECUs) and the recurring lemmas characterizing them, highlighted in italics. Any mention of specific physicians, hospitals, or cities in the text was anonymized and replaced with “Name of physician”, “Name of hospital”, and “Name of city”, respectively.

#### 3.1.1. Cluster 1: “Variability in the Care Experience”

This cluster includes narratives describing the variability of care experiences across different healthcare settings and professionals. It highlights how patients and family carers report markedly different evaluations depending on the specific contexts and encounters they face (*hospital ward*, *healthcare staff*, *nurse*).

While some narratives emphasize trust, gratitude, and relief, others highlight problems, inefficiencies, and dissatisfaction. Lemmas expressing appreciation (e.g., *thank_to_God*, *well*, *excellent*, *to work*, *to find [well/bad]*) co-occur with lemmas indicating criticism and dissatisfaction (e.g., *problem*, *complain*). Verbs such as *to go* and *to send* point to an active search for adequate care, which sometimes entails travelling long distances or changing physicians to obtain better attention. Temporal adverbs (*every time*, *never again*, *always*) indicate a tendency to frame experiences in generalized or absolutized terms. Taken together, these co-occurrence patterns indicate the perceived heterogeneity and unpredictability of care.

The most representative Elementary Context Units (ECUs) can be grouped into three recurring thematic patterns.

First, ECUs describe care experiences as uneven and contingent, with narratives oscillating between trust, gratitude, and dissatisfaction depending on specific encounters.

In the following excerpts, the interviewees contrast different surgical experiences across hospitals and departments, showing how comparable clinical situations can be associated with markedly different outcomes and evaluations of care depending on organizational arrangements and relational encounters.

“A negative *experience* in another surgical *hospital department* where, organizationally, they are just bad and for them my mother was *inoperable*. They *sent* us to oncology, we went up to the second floor to the oncologic centre, we *went* to the admission and the people were helpful and polite, the nurse took everything all.”

(family carer, woman, age range: 51–65, follow up visit, IDnumber_31)

“Because I had *surgery* in NAME OF HOSPITAL, and I was fine. Here, honestly, at NAME OF HOSPITAL, I was not fine. I had a fibroid removed in the gynaecological *hospital department*, and I had a terrible *experience*. Then I *went* home, had a haemorrhage, and had to come back.”

(family carer, woman, age range: 31–50, follow up visit, IDnumber_111)

Second, ECUs report instance of active searching and mobility, involving travelling, switching physicians, or moving across hospitals to manage variability. This mobility is associated with both attempts to cope with fragmented or unsatisfactory care and efforts to preserve relationships of trust with specific physicians.

“It is (a) positive (experience). Even though I live in NAME OF CITY and could easily *go* to NAME OF CITY, because of the trust I have in these physicians and because we have felt comfortable from the beginning, we travel many kilometres, but we prefer to come here to NAME OF CITY”

(family carer, woman, age range: 31–50, follow up visit, IDnumber_18)

“With him we were fine, with my brother it was the situation, my brother was staying here and then he *went* to another oncologist. He’s staying in NAME OF CITY. The colleague, rightly, when she saw him [my father] like this, told him “Have him followed by NAME_OF_PHYSICIAN who is not feeling well. They wanted to go NAME OF CITY, as many people do, too much money. With dad, *thank_to_God*, we got along well.”

(family carer, woman, age range: >65, follow up visit, IDnumber_1)

Third, ECUs frequently refer to lack of continuity with the same physician and organizational variability.

“Discrepancies there were not; I did the visit, in a state that everything was fine, visit done without any major *problems* of note, and I repeat the *problem* is *always* that I did four or five visits and always with different physicians, which in my opinion should be otherwise, so that I always have a relationship with the same person”

(patient, man, age range: >65, follow up visit, IDnumber_76)

#### 3.1.2. Cluster 2: “The Ideal Care Relationship”

This cluster includes narratives referring to participants’ representations of the ideal therapeutic relationship, as well as the perceived gap between expectations and actual experiences. It encompasses narratives describing the characteristics attributed to an “ideal” physician, including not only the ability to address medical problems but also attentiveness to patients’ psychological needs, exploration of emotions, timely communication of bad news, and guidance for family members on how to cope.

At the lexical level, lemmas related to the physician–patient relationship (*patient*, *physician*, *relationship*, *oncologist*) co-occur with those referring to normativity and expectation (*should*, *ideal*) and to the actual experience (*lived*). References to different healthcare contexts (e.g., *Switzerland*, *private care*) further situate these expectations within comparative frameworks. Lemmas expressing the need to be understood and to receive responses (*to understand*, *answer*) co-occur with lemmas emphasizing the importance of what is at stake (*aspect*, *to live*, *life*, *fundamental*) and the expectation that one’s perceived rights will be respected (*to want*, *duty*).

Taken together, these co-occurrence patterns point to the centrality of the care relationship in the corpus as a central domain in which both technical competence and relational qualities are expected to converge.

The most representative Elementary Context Units (ECUs) can be grouped into four recurring thematic patterns.

First, ECUs describe the ideal care relationship in terms of a form of emotional support and recognition, where empathy, attentiveness, and relational closeness are central. Partecipants emphasize the importance of feeling welcomed and acknowledged as persons. In the following excerpt, the physician is expected to provide emotional proximity despite institutional constraints:

“It *should be a relationship* much closer to the patient, in the sense that the physician should be a little bit more empathetic, and, I *understand* that it is difficult because, of course, they see so many patients, so many cases and so many issues; however, the patient has to feel, in a way, welcomed, by the physician, which is not always the case or not for all *physicians*.”

(family carer, woman, age range: 31–50, follow up visit, IDnumber_97)

Second, the ideal physician is defined through technical competence and professional responsibility, ensuring appropriate clinical decisions and providing reassurance through expertise and thoroughness.

“A *physician should* be concerned about the *patient*’s health. So, I expect that because he is a *physician*, he knows the various procedures to be followed so that the *patient* somehow can come out of his problem. I expect him to be a trained physician who is ready to do all the appropriate tests that a *patient* needs.”

(family carer, woman, age range: 31–50, follow up visit, IDnumber_24)

Third, participants articulate the ideal relationship as one of communication, understanding, and recognition of patients’ needs, where being listened to and receiving clear answers becomes essential in relation to existentially significant issues.

“If they are closed and don’t say words, a *patient wants* to know instead a lot of information, so you have to give it to them, then at home you shut up if you don’t *want* to talk.”

(family carer, woman, age range: 51–65, follow up visit, IDnumber_31)

“So, you can give us an *answer* on whether we can help her or whether we *should* let her die like this. But tell us because one organizes, organizes with her mom, takes care of her differently and we make her do something that maybe she has *lived* always working, poor thing. And so, they *should* tell us, they *should* be faster in giving this *answer*, they *should* be faster, just this.”

(family carer, woman, age range: 51–65, first visit, IDnumber_20)

Fourth, ECUs refer to a holistic view of the patient, where emotional and relational dimensions are seen as fundamental and inseparable from clinical care.

“(…), a_times it happens that *physicians* are more concerned about treating only the pathology; instead, the *patient should* be seen as a whole, as a whole, so even the emotional *aspect*, which is *fundamental*, even welcoming with a smile, a_times it happens”

(family carer, woman, age range: 51–65, follow up visit, IDnumber_80)

#### 3.1.3. Cluster 3: “Waiting Times and Delays in Care”

This cluster includes narratives referring to the temporal and organizational dimension of care, with a specific focus on waiting times and perceived delays. It highlights the time spent waiting for care, both in the waiting room before a scheduled follow-up visit or therapy, and for diagnostic tests or results.

At the lexical level, lemmas referring to temporal markers (*minute*, *days*, *months*, *week*, *afternoon*, *time*, *timetable*) co-occur with those related to waiting (*waiting*, *to wait*, *to wait for*, *long*), reflecting the substantial time required to access consultations, diagnostic exams, and treatments (*appointment*, *medical test*, *medical examination*, *therapy*). Action lemmas (*to call*, *to enter*, *to leave*, *to return*) point to the bureaucratic and administrative hurdles patients and family carers encounter in navigating care pathways.

The most representative Elementary Context Units (ECUs) can be grouped into three recurring thematic patterns.

First, ECUs describe waiting as a prolonged and uncertain, often associated with lack of timely communication about clinical processes or diagnostic outcomes, generating anxiety and a sense of vulnerability.

“So, when she told me this, I said OKAY, the physician told me to turn, space so without *waiting* for *appointments*, which then they *called* us back after one *month* and ten *days*. You can’t *leave* the patient *waiting* after one *month* and ten *days*, meet once a *week at least every ten days* and communicate; you can’t keep a person *waiting* who doesn’t know what he has.

(family carer, woman, age range: 51–65, follow up visit” IDnumber_31)

At the same time, waiting is also described as requiring patients to adapt to institutional rhythms, where prolonged delays become normalized and individuals are expected to suspend their ordinary temporal expectations.

“Before doing the *therapy* then you *wait for* the *medical examination* at the reception, and for the infusion, so some *time* passes. I don’t know, for example, this morning I was holding the *medical examination* at 10:20, and I did it 20 *min* ago, that is and *hour* and forty *minutes wait*, and that is not good for a patient”

(patient, man, age range: 31–50, visit therapy, IDnumber_9)

“Because that’s what the *times* are, *time* to *come* in, *time* to talk, then you have to *wait for* the *therapy to come in* and you can also clear the chair to get the infusion done, then you have to do it; so, anyway that’s what the *times* are. Let’s say in the morning and even early *afternoon*, you know when you *come in* here, you have to put your watch aside.”

(patient, woman, age range: 51–65, follow up visit, IDnumber_127)

Second, ECUs refer to delays in reception systems, scheduling practices, and coordination between medical examinations and treatments.

“The only flaw here is *waiting a long time* at the reception, you *wait* a lot, meaning they do not *respect the timetable*. I understand ten, fifteen, twenty *minutes*, even half an *hour*, but sometimes you *wait for* an *hour* and a half, even two *hours* before being *called*, and just as *long* before receiving *therapy*”.

(patient, man, age range: 31–50, visit therapy, IDnumber_4)

Third, ECUs also refer to fragmented care trajectories, where sequential procedures are poorly coordinated and administrative loops prolong uncertainty and disrupt the continuity of care.

“For example, the other *time*, she had to have the PET scan and she couldn’t have the *medical examination* until they gave her the PET scan. She kept the *medical examination;* however, they still did not *call* her to do the PET scan. She came to the *medical examination*, and they said, whatever but without PET? How should I know if they didn’t *call* me. So then again, make another *appointment* again. It’s *long*.”

(family carer, woman, age range: 31–50, follow up visit, IDnumber_111)

#### 3.1.4. Cluster 4: “The Luck of Being Cared by a Good Physician”

This cluster includes narratives describing positive relational experiences with competent, caring, and helpful healthcare professionals, framed not as stable or systematically distributed features of care, but as contingent outcomes of individual encounters.

At the lexical level, adjectives referring to the physician—and more broadly the healthcare staff—are consistently positive, emphasizing competence, availability, friendliness, and a perceived human approach (*good*, *available*, *friendly*, *human approach*). These elements co-occur with lemmas expressing contingency and chance (*difference*, *lucky*, *to find*, *to look for*).

The most representative Elementary Context Units (ECUs) indicate a recurring thematic pattern related to the contingency of positive care experiences. This contingency is articulated through three interrelated discursive manifestations.

First, ECUs describe positive care experiences as linked to encounters with individual physicians. Narratives frequently frame satisfactory experiences as dependent on “luck” and on the possibility of meeting the “right” physician within an otherwise variable system.

“In my opinion, it *always depends* on the people and the physicians you meet because even if they are not all the same and I have to say that I was also *lucky* with my disease because I *found* the surgeon who operated on me for example was very *good*, humanly very *good*.”

(patient, woman, age range: >65, therapy, IDnumber_51)

Second, this contingency is reinforced over time through repeated encounters that are still interpreted as fortunate rather than guaranteed. Even when participants report sustained positive experiences, these are not attributed to continuity in care structures, but to the recurrence of “good luck” in meeting supportive professionals.

“We are lucky that *good* physicians were *always found*, and we felt comfortable. So, we are lucky on this side.”

(family carer, woman, age range: 51–65, follow up visit, IDnumber_17)

Third, the coexistence of positive and negative professional encounters across care trajectories further strengthens the perception of variability as an inherent feature of the system.

“in the course of the disease we have *found good* physicians and oncologists, as we have *found* the figure I *look for*, so the one who keeps me most alive and motivated. But I have also *found* the opposite. I have often confronted and clashed with physicians.”

(family carer, man, age range: 51–65, follow up visit, IDnumber_34)

“In my opinion you need the physician, but you also need the *human* approach with the patient. They referred him to us as a physician of excellence medically, however, …who you were *talking* to, this character is just his, this way, I *talk* about this physician. For example, then here at the oncology we *found* the physician *good* and *available*”

(family carer, woman, age range: 51–65, follow up visit, IDnumber_29)

### 3.2. Relationship Between Respondents’ Characteristics and Clusters

As shown in Table 5, Correspondence Analysis (CA) indicated no significant differences in cluster distribution by respondents’ role (patient vs. family carer) or sex. Both patients and family carers, in fact, shared similar semantic repertoires centred on waiting, uncertainty, and variability of care experiences.

Significant differences emerged, instead, for age and reason for being in the hospital. Specifically, younger respondents (18–30 years) were underrepresented in Cluster 1 and overrepresented in Cluster 2; respondents aged 31–50 years were overrepresented in Cluster 1, while those aged 51–65 were underrepresented. Participants attending the hospital for a first visit were underrepresented in Cluster 1, whereas those receiving treatment were overrepresented.

## 4. Discussion

This cross-sectional study explored how cancer patients and family carers describe their expectations and experiences of care at a Local Hospital in Southern Italy. Cluster analysis identified four semantic cores reflecting key aspects of the care experience, revealing how these are deeply shaped by organizational arrangements, resource distribution, and the institutional conditions that enable or constrain relational continuity, communication, and trust within healthcare encounters.

In Cluster 1 (Variability in the care experience)—where respondents aged between 31 and 50 years and waiting in the hospital for treatment are overrepresented—satisfaction or dissatisfaction with care were closely related to the perceived competence and attentiveness of encountered specialists. While many participants expressed appreciation for physicians, they emphasized marked variability in care quality, encapsulated in the perception that “physicians are not all the same.” The absence of stable relationships with the same physician is experienced as an additional layer of unpredictability (Kern et al., 2019).

Ensuring appropriate care often required changing doctors or travelling long distances, reflecting structural inequalities within the Italian healthcare system. These findings are consistent with previous research highlighting persistent regional disparities in access to and quality of care. Healthcare services in Southern regions of Italy are commonly perceived as lower in quality than those in the North, particularly in terms of treatment standards and waiting times (Greco, 2025; Toth, 2014). In this context, the so-called *viaggi della Speranza*—journeys undertaken to seek cancer care in Northern Italy—remain widespread (Greco, 2019), illustrating how geographical mobility becomes an informal strategy to compensate for territorially uneven provision of care. Findings also resonate with qualitative research conceptualizing cancer survivorship as a process of “wrangling” with healthcare systems, understood as the everyday struggle to navigate complex, uneven, and sometimes unwelcoming institutional pathways (Dew et al., 2024). Recent studies have further framed these challenges as barriers related to access, care coordination, and systemic complexity, requiring patients to actively manage delays, fragmentation, and administrative obstacles across the cancer care pathway (Chen et al., 2024; Hoeve et al., 2026). The present study extends this perspective by showing that such efforts are not only directed at navigating the system, but also at securing trustworthy and reliable relational engagement with physicians. In this sense, variability in care emerges as both a structural and relational phenomenon, shaping inequalities in the quality of patient–physician relationships. Active strategies of mobility therefore represent not only a pragmatic response to uneven service provision, but also a relational strategy aimed at maintaining therapeutic trust.

Cluster 2 (The ideal care relationship), where respondents aged between 18 and 30 years are overrepresented, emphasizes expectations of physicians who attend not only to disease but to patients as biopsychosocial beings, acknowledging fears, anxieties, and concerns (Busch et al., 2019). Participants highlighted empathy, respect, compassion, and attentive listening, viewing technical competence as necessary but insufficient. Relational and experiential aspects of care frequently emerge as major sources of dissatisfaction and complaint across health care setting (Mattarozzi et al., 2017; Reader et al., 2014; Venuleo et al., 2024). In Italy, where the present study is situated, complaints most commonly concern care system management—particularly waiting times—and relational aspects of care, reported in over half of cases (Mattarozzi et al., 2017). Users consistently report valuing respect, empathy, and attentiveness in their interactions with healthcare professionals (Venuleo et al., 2024). Our findings confirm and further articulate this evidence by showing that the “ideal” care relationship is not defined by a single dimension, but by the convergence of multiple relational expectations, which can be analytically distinguished into four interrelated components.

First, the relationship is constructed as a form of emotional support and recognition, where feeling welcomed, acknowledged, and treated as a person becomes central. This finding is consistent with previous research on patient-centred care but adds nuance by showing how such expectations explicitly emerge in contrast with experiences perceived as emotionally distant or routinized. Second, participants emphasize technical competence and professional responsibility as integral to the ideal relationship, highlighting that clinical expertise remains a necessary condition for trust. Here, expectations focus on what physicians are able to do—namely, making appropriate clinical decisions, ensuring accurate diagnoses, and guiding the care process effectively. In line with existing literature, competence is not opposed to relational qualities but expected to coexist with them, reinforcing the idea that patients evaluate care through a combined clinical–relational lens rather than along separate dimensions. Third, the relationship is framed as a communicative process grounded in understanding, responsiveness, and the provision of clear answers, particularly in relation to existentially significant issues such as prognosis and end-of-life decisions. Physicians were expected to support family members and to communicate bad news in a timely and sensitive manner, consistent with previous findings (Primeau et al., 2024; Gonella et al., 2019; Venuleo et al., 2022). Although transparent communication supports emotional adjustment and decision-making, physicians may withhold or filter information due to concerns about patients’ emotional reactions or uncertainty about how to convey difficult news (Berkey et al., 2018). Consistently, Legese et al. (2021) reported that many breast cancer patients perceived the information received as insufficient, contributing to distress and uncertainty. Our findings extend this literature by showing that unmet communicative expectations are not only informational gaps but are experienced as failures of recognition and responsiveness within the relationship. Fourth, a holistic view of the patient emerges as a distinct and complementary component of the ideal relationship, where emotional and relational dimensions are seen as fundamental and inseparable from clinical care. Rather than referring to what physicians are expected to do in terms of clinical performance, this dimension concerns how patients expect to be understood and positioned within the care process—not merely as carriers of a disease, but as whole persons with emotional, relational, and existential needs. While this aligns with the biopsychosocial model widely advocated in the literature, our findings highlight how patients and caregivers actively mobilize this model as an evaluative standard to assess the adequacy of care.

Cluster 4 (The luck of being cared by a good physician) highlights how participants often describe very positive experiences with physicians, who are portrayed as exceptionally kind, attentive, and humane. The physician is idealized: he or she is not merely a professional fulfilling a duty, but an exceptional figure, deserving of mention and gratitude. This idealization and full confidence in the physician can be understood, on one hand, as a cognitive process—first needing to trust the physician and then preserving the consistency of one’s beliefs by assuming the physician possesses the qualities required to warrant that trust—and, on the other hand, as an emotional stance to face the uncertainty of the health condition and gain a subjective sense of safety. Notably, when the actual relationship matches the ideal, it is narrated as a matter of “luck” rather than as an expected outcome of a well-functioning healthcare system. In line with the results, this sense of contingency is not limited to isolated encounters but is reproduced over time, as repeated positive experiences are still interpreted as fortunate rather than as indicators of systemic reliability. Such narratives implicitly normalize systemic failures by reframing adequate care as exceptional. One reason for this perception is the lack of continuity in care: physicians may change at each visit, preventing stable relationships. Previous research indicates that regularity of contact is more important than frequency, particularly for patients facing uncertainty (Dyer et al., 2022). In Italy, the context of the present study, the lack of regularity must also be framed in the light of a health system suffering from a progressive decrease in resources allocated for public health, characterized by insufficient availability of medical personnel, as well as of products and physical structures. The perception that empathic care is not the norm is also consistent with research on healthcare complaints, which shows that dissatisfaction often concerns relational rather than technical aspects of care (Mostafapour et al., 2024a, 2024b). Physicians may underestimate communication difficulties and patients’ emotional distress (Gössi et al., 2025), and communication failures remain a frequent source of complaints (Alamo-Palomino et al., 2020; Kee et al., 2018; Mack et al., 2017). Despite its recognized benefits, patient- and family-centred care remains insufficiently embedded in routine practice, constrained by biomedical models, professional norms favoring emotional detachment, time pressures, and organizational demands (Halpern, 2014; Martínez-Morato et al., 2021). Physicians may fear that engaging emotionally with patients is time-consuming, yet evidence shows that allowing patients to speak freely requires minimal time and enhances satisfaction and adherence (Langewitz et al., 2002). At the same time, structural stressors—such as staff shortages, heavy workloads, the high-pressure environment of oncology departments, and limited managerial support—further heighten the risk of burnout and reduce empathic engagement (Ashouri et al., 2018; Holland, 2010).

Cluster 3 (Waiting Times and Delays in Care) focuses on waiting as a central dimension of care experience. Time spent in waiting rooms was described as “suspended” time, requiring patients and carers to set aside work and family commitments. Previous studies show that prolonged waiting marginalizes everyday life in favour of illness-related demands (Hall et al., 2021; Paul et al., 2012). Participants reported waiting times of several hours, consistent with earlier findings (Bielen & Demoulin, 2007; Venuleo et al., 2024; Xie & Or, 2017). Delays in appointments and diagnostic results align with OECD data identifying long waiting times as a major policy concern (OECD, 2025) and is particularly pronounced in Southern Italy (ISTAT, 2023). Such delays are associated with distress, uncertainty, and reduced satisfaction, whereas shorter waits enhance perceptions of staff compassion (Lamba et al., 2020; Liddy et al., 2024; Söderlund, 2023).

While these findings confirm the well-documented impact of waiting times on patients’ experiences, the present study further nuances this evidence by showing that waiting is not only a quantitative issue (i.e., duration), but also a qualitative and organizational one. First, consistent with the results, waiting is experienced as a lived temporal burden, characterized by prolonged uncertainty and emotional strain, particularly when communication about clinical processes and diagnostic outcomes is lacking or delayed. Second, waiting is described as requiring patients to adapt to institutional temporalities, where delays are normalized, and individuals are expected to suspend their ordinary temporal expectations. This dimension suggests a process of accommodation to organizational rhythms that has been less explicitly addressed in prior literature. Third, waiting is embedded in fragmented care trajectories, where sequential procedures are poorly coordinated and administrative loops prolong uncertainty. This finding adds to existing evidence by highlighting that waiting is experienced as a cumulative and systemic process, rather than as a series of isolated inefficiencies.

No significant differences emerged between patients and family carers in the distribution of semantic cores, indicating shared expectations and interpretative frameworks. This convergence suggests that cancer is experienced as a relational condition, characterized by jointly negotiated meanings, emotional attunement, and shared vulnerability (Y. Wang & Feng, 2022). Both groups emphasized uncertainty, waiting, and variability in care, pointing to common experiences of temporal disruption, organizational challenges, and emotional strain. Carers’ narratives closely mirrored those of patients, particularly regarding empathic communication, continuity of care, and timely access to services. Rather than indicating a lack of specificity in carers’ experiences, this alignment highlights a shared meaning-making context within the care relationship, reinforcing the relevance of a family-centered perspective in which patients and carers are understood as a relational unit interacting with the healthcare system.

### 4.1. Theoretical Contribution

This study makes several contributions to the literature on care relationships in oncology. First, it identifies a shared experiential and semantic framework between patients and family carers, showing that expectations, evaluations, and meanings of care are co-constructed within a relational context rather than being confined to individual perspectives. Second, it advances current understanding of care relationships by conceptualizing them as inherently ambivalent: while individual encounters with healthcare professionals are often experienced as supportive and reassuring, they are simultaneously embedded in organizational conditions characterized by variability, discontinuity, and delays, which generate tension and uncertainty. Third, by applying semantic cluster analysis to narrative data, the study demonstrates the added value of this methodological approach in capturing the coexistence of relational and structural dimensions of care, making visible patterns of meaning that might remain implicit in more traditional qualitative analyses. Taken together, these contributions provide a more integrated account of how care is experienced, interpreted, and negotiated within oncology settings.

### 4.2. Practical Implications

The present findings provide several actionable insights for clinical practice and healthcare organization, highlighting strategies to enhance patient–caregiver experiences across relational, temporal, and systemic dimensions.

First, the variability in care quality and relational experiences highlighted in Cluster 1 underscores the importance of continuity and relational consistency. Clinicians and healthcare managers should prioritize strategies that foster stable patient–physician relationships, minimizing the need for patients to actively search for trusted providers (Dew et al., 2024; Chen et al., 2024). Ensuring that patients and carers can access the same physician across visits can reduce perceived and enhance both satisfaction and adherence to treatment (Dew et al., 2024).

Second, expectations around the ideal care relationship (Cluster 2) indicate that empathy, attentive listening, and recognition of psychosocial needs are as critical as technical competence. Training programs emphasizing communication skills, emotional attunement, and timely delivery of sensitive information can enhance patient satisfaction and support carers’ understanding (Primeau et al., 2024; Venuleo et al., 2022). Providing structured guidance for family members on coping strategies can also strengthen the relational dimension of care (Gonella et al., 2019).

Third, prolonged waiting times and delays (Cluster 3) emphasize the need for proactive temporal management and clear communication. Providing realistic timelines for diagnostic procedures and treatment steps, alongside regular updates for both patients and carers, can reduce anxiety, uncertainty, and the emotional burden associated with waiting (Hall et al., 2021; Liddy et al., 2024). Tools such as appointment reminders, written summaries, or dedicated care coordinators can support families in navigating complex pathways efficiently.

Fourth, the contingency of positive experiences (Cluster 4) underscores that empathic care should be systemically embedded rather than left to chance. Organizational policies should actively monitor and support relational quality across wards, including feedback mechanisms for patients and carers and protocols for ensuring consistency in staff behavior and availability (Dyer et al., 2022; Mostafapour et al., 2024a). Recognizing and reducing the role of “luck” in positive experiences can normalize empathic care as a standard expectation rather than an exceptional outcome.

Finally, the convergence between patients’ and carers’ narratives suggests that interventions should target the patient–caregiver dyad as a unit. Psychosocial support programs, information sessions, and counseling should involve both parties, recognizing their shared vulnerabilities and jointly negotiated meanings around care (Legese et al., 2021; Y. Wang & Feng, 2022).

Together, these recommendations advocate for a holistic, family-centred approach that integrates structural improvements, relational continuity, and psychosocial support, thereby addressing the multifaceted challenges revealed by patients’ and caregivers’ narratives.

### 4.3. Limitations

This study has some limitations. First, it was conducted in only one oncology department in southern Italy, and the results are not generalizable. Results may vary depending on culture and context. Second, no data were collected on the characteristics of those who declined participation, which could represent a potential selection bias. For example, it is possible that those who accepted the invitation to participate in the study were mainly the patients and family members who were most satisfied with their experience of care. Third, the cross-sectional design provides a snapshot of experience; longitudinal studies are needed to examine changes over time and the role of socio-demographic (e.g., educational level, social status) and clinical (e.g., indication, type and amount of treatment) characteristics that could be a source of variation in the perceived quality of the interaction.

## 5. Conclusions

This study provides insights into dimensions of cancer care experience that traditional quality and safety indicators often fail to address (Råberus et al., 2019; Reader et al., 2014). Leveraging automated semantic analysis, it systematically identifies patterns of meaning across patients’ and carers’ narratives, offering a nuanced and data-driven understanding of cancer care experiences that extends beyond what traditional qualitative approaches typically capture. By including both patients and family carers, the study uniquely illuminates the experiences of all key actors in the care process and shows how expectations and evaluations of the care relationship are jointly constructed.

Although participants generally report positive experiences and express gratitude toward physicians, they also describe care quality as precarious and contingent, marked by variability, lack of continuity, and delays in diagnosis and treatment, fostering the perception that satisfactory care depends on chance rather than on a reliable healthcare system. Importantly, relational experiences in oncology emerge as inherently ambivalent: they are experienced as supportive and reassuring in interactions with individual clinicians, while simultaneously being tension-laden when framed against systemic and organizational constraints such as waiting times, fragmented care pathways, and discontinuity of care. These dual dimensions are consistently reflected in participants’ narratives, highlighting the complex interplay between personal encounters and structural conditions in shaping how patients and caregivers interpret, navigate, and evaluate healthcare experiences.

The convergence between patients’ and carers’ narratives indicates shared emotional burdens, interpretative frameworks, and vulnerabilities, which shape their expectations and evaluations of clinical encounters. This suggests the need for a patient–family integrated approach in which healthcare interventions, communication strategies, and psychosocial support engage both patients and carers as interdependent unit within the care process.

However, promoting compassionate and family-centred care cannot rely solely on individual physicians’ skills. Institutional and policy-level actions aimed at improving resources, staffing, and continuity of care are equally crucial. As shown during the COVID-19 emergency, organizational pressures and resource shortages contribute to burnout among healthcare workers (Marinaci et al., 2020, 2023), undermining professionals’ capacity to provide relationally attuned care. Ultimately, ensuring equitable, high-quality cancer care requires addressing the broader structural conditions shaping both patient vulnerability and providers’ responsiveness. Relational quality, communication, and continuity are not optional “soft” dimensions of care but core elements of health system performance that demand strategic investment and governance.

## Figures and Tables

**Table 1:** Interview guide—Open-ended questions used in the interviews.

How would you describe the ideal relationship between physician and patient? (*Elicits participants’ expectations and values regarding the doctor-patient relationship.*)
Think about the last visit you made to this hospital. How would you describe the quality of the relationship between you and the physician? (*Encourages participants to reflect on their most recent lived experience.*)
From your point of view, on whom or what does the difference that sometimes exists between an ideal doctor–patient relationship and the actual relationship depend? (*Explores perceived barriers and enablers affecting relational quality in healthcare settings*.) More broadly, how would you describe your relationship with the healthcare staff you have met in this hospital? (*Extends the focus from the doctor-patient relationship to the wider healthcare system*.)
Is there anything else you would like to add? (*Allows participants to express concerns or insights that may not have emerged through the previous questions.*)

**Table 2:** Descriptive Characteristics of participants.

Variables		Patients (*n* = 57)	Family Carers (*n* = 86)	Total (*N* = 143)	χ^2^	*p*-Value
Sex	Men	22 (38.6%)	22 (25.6%)	44 (30.8%)	2.73	0.10
	Women	35 (61.4%)	64 (74.4%)	99 (69.2%)		
Age range *	18–30	0 (0.0%)	5 (3.7%)	5 (6.3%)	10.97	0.01
	31–50	10 (18.2%)	25	35 (31.3%)		
	51–65	24 (43.6%)	36	60 (45.0%)		
	>65	21 (38.2%)	14	35 (17.5%)		
Role with respect to the patient	Son/Daughter		41 (47.7%)	41 (47.7%)		
	Brother/Sister		6 (7.0%)	6 (7.0%)		
	Spouse/Partner		24 (27.9%)	24 (27.9%)		
	Other Relative or Friend		15 (17.4%)	15 (17.4%)		
Reason why the respondent was in hospital **	First visit	7 (12.3%)	12 (8.6%)	19 (13.3%)	1.26	0.53
	Therapy	3 (5.3%)	9 (6.4%)	12 (8.4%)		
	Follow-up	45 (78.9%)	64 (45.7%)	109 (76.2%)		

* Percentages calculated on valid cases (Patients *n* = 55; Family carers *n* = 80; Total *N* = 135). Eight responses were missing (2 patients, 6 family carers). ** Percentages calculated on valid cases (Patients *n* = 55; Family carers *n* = 85; Total *N* = 140). Three responses were missing (2 patients, 1 family carers).

**Table 3:** Dataset.

	*n*
Texts in the corpus	143
Elementary contexts (EC)	1538
Types	6435
Occurrences (Tokens)	73,585
Threshold of lemma selection	10
Lemmas in analysis	448

Note—Texts in the corpus: number of answers to the open question (corresponding to the number of participants) inserted in the text analysis; Elementary context: sections of text (e.g., sentences, paragraphs, or short texts) characterized by the same keyword patterns; Types: total number of words (i.e., including all linguistic forms) contained in the general corpus; Occurrences (Tokens): frequencies of a single lexical unit; Threshold of lemma selection: the value selected to include the lemma in the analysis; Lemmas in analysis: number of headwords inserted in analysis.

**Table 4:** Key lemmas characterizing the clusters.

Cluster 1 (25.6%)	χ^2^	Cluster 2 (32.33%)	χ^2^	Cluster 3 (18.5%)	χ^2^	Cluster 4 (23.6%)	χ^2^
Hospital ward	192.47	Should	317.70	Waiting	116.18	Good	192.44
Well	121.51	Relationship	55.65	To Wait	90.54	Available	135.11
Problem	82.98	Patient	48.48	Minute	80.99	To Find	47.68
To find [well/bad]	76.68	Ideal	27.81	Medical_test	54.55	Always	46.97
To go	65.68	Lived	23.30	Days	49.87	To look for	36.30
To send	38.37	Answer	18.77	To Call	45.17	Young	33.77
Excellent	31.97	To live	18.57	To come in	44.54	Positive	33.31
Chemotherapy	29.71	Physician	18.37	Medical_examination	38.43	HCS *	29.92
Thanks_to_God	29.62	To Think	18.30	Month	35.48	To Prepare	27.99
Never again	28.52	Life	16.78	Afternoon	33.81	Father	27.51
Every time	26.62	Manner	16.70	Timetable	29.05	To Talk	27.18
Against	21.00	To Understand	16.47	Week	25.61	Experience	26.49
To complain	18.67	Oncologist	15.69	Time	25.46	HCS **	25.80
Experience	18.25	Fundamental	15.38	To return	24.38	To see	24.60
Request	17.74	To want	15.20	Long (wait)	22.90	To Depend	21.42
Healthcare_staff	17.53	To stand	13.61	To respect	21.70	Our	19.99
Nurse	16.99	Aspect	13.25	Appointment	21.41	HA ***	18.85
Always	14.65	Duty	13.24	To leave	20.07	Friendly	17.96
To work	13.69	Private care	13.17	Therapy	19.35	Difference	16.50
To operate (surgically)	13.23	Switzerland	12.70	To wait for	19.24	Lucky	15.79

* Healthcare_system; ** Healthcare_staff; *** Human_approach.

**Table 5:** Significant Associations Summary.

Variable	Cluster 1: Variability in the Care Experience	Cluster 2: The Ideal Care Relationship	Cluster 3: Waiting Times and Delays in Care	Cluster 4: The Luck of Being Cared by a Good Physician
Target				
Patient	−1.31	0.58	0.84	−0.06
Parent	1.31	−0.58	−0.84	0.06
Sex				
Women	0.39	−0.99	−0.78	1.40
Men	−0.39	0.99	0.78	−1.40
Age Range				
18–30	−2.06 *	2.16 *	−0.72	0.39
31–50	2.84 *	−0.40	−0.60	−1.93
51–65	−3.33 *	0.92	0.52	1.93
>65	0.60	−0.43	−0.47	0.28
Reason for visit				
First visit	−2.93 *	0.28	1.55	1.29
Therapy	4.23 *	−1.83	−0.89	−1.51
Follow-up	−0.28	1.06	−0.92	−0.06

Test-values above ±1.96 indicate a statistically significant association between category and cluster. Positive values indicate that the mode is more present in the cluster than the other modes, negative values indicate that the mode is underrepresented in the cluster. * indicates statistically significant associations (*p* < 0.05).

## Data Availability

The dataset is qualitative and contains several quotes that could potentially identify participants. Therefore, the raw dataset will not be available. However, additional quotes supporting each theme can be provided upon request from the corresponding author.
